# Indirect Regeneration and Assessment of Genetic Fidelity of Acclimated Plantlets by SCoT, ISSR, and RAPD Markers in* Rauwolfia tetraphylla* L.: An Endangered Medicinal Plant

**DOI:** 10.1155/2019/3698742

**Published:** 2019-04-11

**Authors:** Gulab Khan Rohela, Phanikanth Jogam, Prasad Bylla, Christopher Reuben

**Affiliations:** ^1^Department of Biotechnology, Kakatiya University, Warangal 506 009, Telangana, India; ^2^Biotechnology Section, Moriculture Division, Central Sericultural Research & Training Institute, Central Silk Board, Ministry of Textiles, Government of India, Pampore 192 121, Jammu and Kashmir, India; ^3^Department of Botany, Kakatiya University, Warangal 506 009, Telangana, India

## Abstract

*Rauwolfia tetraphylla* L. is an important medicinal plant species which is well known for its pharmaceutically important alkaloids. In the present study, we are reporting about its conservation by* in vitro* clonal multiplication through the standardized protocol of indirect regeneration by using leaf and stem based callus and assessment of genetic fidelity of acclimated plantlets by start codon targeted (SCoT), inter simple sequence repeats (ISSR), and randomly amplified polymorphic DNA (RAPD) marker based analysis. Initially friable callus was induced in maximum amounts (378.7, 323.8, and 412.8 in mg) from leaf, root, and stem explants on Murashige and Skoog (MS) media supplemented with 5.0 mg/L, 3.0 mg/L of 2,4-dichlorophenoxyacetic acid (2,4-D) and 5.0 mg/L of naphthalene acetic acid (NAA), respectively. Shoot regeneration with the maximum number of shoot buds (25 and 20) was obtained from leaf and stem calluses on MS media supplemented with TDZ (0.25 mg/L) + BAP (2 mg/L). The regenerated shoots were rooted successfully with maximum rooting percentage of 98.0 on full strength MS media amended with IAA (1.0 mg/L) and IBA (1.0 mg/L). The regenerated plantlets were hardened using 2:1 ratio of sterile garden soil and sand, followed by acclimatization in field conditions with 86% of survival. SCoT, ISSR, and RAPD primers based polymerase chain reaction (PCR) analysis was carried out to check possible genetic variations in micro propagated plants in comparison with mother plant. Among the ten SCoT (S), ISSR (R), and RAPD (OPA) primers used, S2, R10, and OPA3 has given good amplification with scorable DNA bands. The results revealed that the regenerated plants did not have any polymorphism with mother plant. Hence, the* in vitro *regenerated* R. tetraphylla* plantlets were confirmed as true-to-type.

## 1. Introduction

In India, Ayurveda is a traditional medicinal system which has been practiced for thousands of years and remains as one of the most ancient and yet living traditions across the country, which has a sound philosophical and experimental basis [1, 2]. India is also the largest producer of medicinal herbs and hence appropriately called as the Botanical Garden of the World [3]. Some of these medicinal plants have been featured on Indian postage stamps, where the first set of stamps came out in 1997, showing four different medicinal plants: Ghritkumari (*Aloe barbadensis*), Sarpagandha (*Rauwolfia serpentina*), Tulsi (*Ocimum sanctum*) and Haridra (*Curcuma longa*) [4] followed by another set of four medicinal plants: Brahmi (*Bacopa monnieri*), Guggulu (*Commiphora wightii*), Amla (*Emblica officinallis*), and Aswagandha (*Withania somnifera*) as postal stamps were released [5].

Presently, medicinal plants play a very important role in the modern economy of developing countries. In India, the medicinal plant related trade is estimated to be approximately US $ 1 billion per year [6]. Recent trends have indicated further increase in this trade with the herbal cosmetic and pharmaceutical industry playing a major role in fulfilling the demand for herbals worldwide. In addition to the international trade, there is a substantial volume of internal trade for medicinal plants in India, which has created a lot of demand to these medicinal herbs and shrubs [7].

Among the different plant families, Apocynaceae has gained lot of medicinal importance, as most of the plant species of this family produce milky sap which has pharmaceutical applications such as for gastrointestinal ailments, malaria, pain and diabetes [8]. Almost every part (roots, stem bark, leaves and latex) of Apocynaceae family members was used for several treatments and disorders like leprosy, dysentery, worms, ulcers, tumours, skin, and liver diseases [9].

*Rauwolfia* is an important genus in Apocynaceae family which has gained much popularity due to the presence of pharmaceutically significant alkaloids with higher concentrations in roots and stem bark. Approximately 130 species of* Rauwolfia* have been identified and are located mostly in tropical and semitropical regions. Among the different species,* R. serpentina* Benth. ex Kurz is indigenous to India [10, 11] and this species is on the verge of extinction [12] due to its overexploitation by ayurvedic practitioners and pharmaceutical industries [13] and Government of India has also restricted the exploitation of this endangered species [14]. In this scenario the attention of industrialists and medicinal practitioners was diverted to find an alternative plant species as a source of pharmaceutical alkaloids.

Among the other species of* Rauwolfia*, the most important is* Rauwolfia tetraphylla *L., which has gained lot of importance as an alternative source of reserpine and other alkaloids [15].* R. tetraphylla *is native of tropical America and West Indies [16], but now it is naturalized [17] throughout the tropics which includes the moist deciduous forests of Southeast Asia including Burma, Bangladesh, India, Malaysia, and Srilanka [18]. The common names are wild snake root plant, devil pepper, be-still tree, American serpent wood, devil root, milk bush etc. It is being cultivated widely as both an ornamental and a source of pharmaceuticals. This species produces different type of pharmaceutically important alkaloids in their stem bark and roots such as reserpine, deserpidine, ajmalicine, ajmaline, canescine, yohimbine, and recinnamine [19, 20].

Due to presence of various commercial indole alkaloids, this plant species was also exploited during the last three decades and presently it is in endangered status [21]. Propagation of* R. tetraphylla* through vegetative cuttings and seed material is limited (5-10%) due to its poor rooting ability and due to presence of phenolic compounds in seed [22]. In view of the above facts about the mass exploitation of this plant from natural resources and due to its restricted cultivation, there is an urgent need to conserve this medicinally important plant species by proper conservation strategy. Hence, the present study is undertaken with the objective of mass propagation of* R. tetraphylla* through the standardized indirect regeneration protocol by utilizing leaf and stem based callus and to confirm the genetic fidelity of acclimated plants by using SCoT, ISSR, and RAPD primer based PCR analysis.

Even though there are some reports about its multiplication through nodal, shoot tip cultures [23] and* in vitro* flowering [24], but there is scanty information about its regeneration through callus based indirect regeneration protocols. In our previous report, we have used single type of cytokinin (TDZ) in regenerating the low frequency of multiple shoots from leaf and stem based callus [25]. Now, in this study we are reporting a protocol for its conservation through high frequency of plantlet production by using combination of cytokinins amended media.

## 2. Materials and Methods

In this study, most of materials and methods were followed as per the report on indirect regeneration studies in a* Morus *species [26].

### 2.1. Plant Material and Explant Source

Two-year-old* R. tetraphylla* plants available in the medicinal arboretum, Department of Biotechnology, Kakatiya University, were used as parent plant material in this* in vitro* propagation studies. Fully expanded leaf of about 7 cm in length and 4 cm in breadth, stem (internodal segments), and root parts were collected during the second week of June and were used as explants for the study.

### 2.2. Murashige and Skoog Media Preparation

MS medium [27] was prepared by using sucrose (3%) and appropriate quantities of vitamins, Iron, macronutrients, and micronutrients from stock solutions. Before dispensing the prepared medium to culture tubes, required concentrations and combinations of plant growth regulators were added to it from the hormonal stock solutions. The media was solidified with 0.8% Agar and pH was adjusted to 5.6 with 1 or 2 drops of 0.1 N HCl or NaOH solution before autoclaving at 121°C for 15 minutes.

### 2.3. Aseptic Techniques for Sterile Transfer

All operations including transfer and sterile filtration were made under laminar airflow cabinet. Prior to operation, the laminar airflow bench was swabbed with 70% alcohol to maintain maximum sterility. The laminar airflow was switched on and kept on UV mode for 30 minutes before using it. The scalpel, forceps and spatula were sterilized by flaming with alcohol before using the laminar airflow cabinet.

### 2.4. Explant Surface Sterilization

Leaf, stem (internodal), and root explants of* R. tetraphylla* were collected from the medicinal arboretum and initially washed under running tap water for 2 minutes. Then different protocols were followed for the sterilization of leaf, stem and root explants, as leaf explants are more delicate and sensitive in nature to sterilants and due to greater degree of fungal count on stem and root explants. Leaf explants were treated with Tween-20 solution for 1 minute followed by 60% ethanol for 2 minutes and in 0.1% mercuric chloride (HgCl_2_) solution for 2 minutes. Stem and root explants were treated initially with 0.5% bavistin for 10 minutes, followed by Tween-20 solution for 2 minutes and with 0.1% mercuric chloride solution for 4 minutes. After every treatment explants were rinsed in sterile distilled water for 2 minutes to remove the adhering chemical sterilants.

### 2.5. Inoculation and Incubation

Surface sterilized leaf, stem and root explants were blotted dry on sterile tissue paper and inoculated onto MS medium under aseptic conditions of laminar air flow cabinet with the help of sterile forceps and culture containers were immediately closed. During inoculation, the test tubes and flasks containing solid medium were held horizontally; this strongly reduces the contaminations. Culture tubes with inoculated explants were kept in culture room under controlled conditions of 16/8 hours of photoperiod with 3000 lux intensity light at 27°C. For each treatment, 25 replicates were used and each experiment was repeated thrice.

### 2.6. Callus Induction

For callus induction, fully expanded leaf, stem (inter nodal), and root explants of* R. tetraphylla *(two years old) were inoculated on to MS medium supplemented with 2,4-D or NAA or Kn (1.0-5.0 mg/L). Data on callus induction from three replicates were recorded at the end of 3 weeks of culture. Each replicate consists of 25 cultures.

### 2.7. Determination of Fresh and Dry Weight of Callus

For determination of fresh and dry weight of callus, 10 identical culture tubes were used. The mean value of each callus was determined. After determining the callus weight, they were dried in an oven at 60°C for 1 hour and average dry weight of the callus was determined. The average fresh and dry weight of the callus was subtracted from the respective callus culture to find out the average gain in fresh and dry weight of the callus. Fresh and dry weights of the callus were noted with a gap of 22 days.

### 2.8. Shoot Regeneration

*In vitro *shoot regeneration was carried from the friable callus by using individual and combinations of cytokinins (BAP and TDZ) supplemented MS media. While preparing combinational cytokinin media, BAP of 2 mg/L was kept constant across all the combinations with altering concentrations of TDZ in low amounts (0.1-1.0 mg/L). Data on number shoot bud regeneration from friable callus in three replicates was recorded at the end of 2nd week of culture. Each replicate of regeneration studies consists of 10 cultures.

### 2.9. Rooting of the Regenerated Shoots

The regenerated shoots were separated from the culture tubes and transferred onto MS medium containing various concentrations (0.5–2.0 mg/L) of individual (IAA or IBA) as well as combination (IAA+IBA) of auxins. Data on root induction from shoot lets of three replicates was recorded at the end of 2nd week of culture. Each replicate consists of 10 cultures.

### 2.10. Hardening and Acclimatization of Rooted Plantlets

Rooted plantlets of* R. tetraphylla *were separated carefully from the culture tubes along with the media and the adhered medium was removed gently by washing under running tap water and then hardened in a plastic pot or plastic cup by using 2:1 ratio of sterile garden soil and sand.

### 2.11. DNA Isolation and Genetic Fidelity Analysis

Genomic DNA was isolated from acclimated plantlets of* R. tetraphylla *(seven plants were selected randomly, four from leaf callus based and three from stem callus based regenerants) from field along with the mother plant. Fresh leaf tissue (500 mg) of both mother plant and* in vitro* regenerated plants was used as raw material for DNA isolation by following modified cetyl trimethyl ammonium bromide (CTAB) method [28, 29]. The quality parameters of DNA were checked by spectrometric and electrophoretic methods.

For testing the genetic homogeneity of regenerated plantlets with that of mother plant, three types of primers (RAPD, ISSR, and SCoT) were used. A total of 30 primers were used,* i.e.*, SCoT (10 primers), ISSR (10 primers), and RAPD (10 primers) for genetic fidelity studies. PCR amplifications were performed using a total volume of 50 *μ*l containing 100 ng DNA, 1X PCR master mix (GCC biotech), and 10 p mole primer. The amplification reaction was carried out in a thermal cycler (Eppendorf, Germany) for 30 cycles with an initial denaturation at 94°C for 5 minutes, followed by denaturation at 94°C for 30 seconds, and annealing was carried at 50°C for 45 seconds for SCoT and ISSR and at 37°C for 45 seconds for RAPD primer based analysis, followed by extension at 72°C for 2 minutes and final extension at 72°C for 5 minutes. After completion of cycles, amplified products were allowed to cool down at a holding temperature of 4°C. PCR products were then separated on 1% agarose gel electrophoresis by using TAE (Tris acetic acid EDTA) buffer. The size of amplicons was estimated using 1kb DNA ladder (Thermo Scientific). All amplification reactions with RAPD, ISSR, and SCoT primers were repeated at least three times to check the reproducibility. The gels were photographed using gel documentation system and only clear and scorable DNA bands were considered for analysis.

### 2.12. Statistical Analysis

The data obtained in this research study was statistically analyzed by using one-way ANOVA in SPSS Version 17 (SPSS Inc., Chicago, USA) and means were compared using Tukey's tests at the 5% level of significance. All means are presented with ± Standard Error (SE).

## 3. Results and Discussion

### 3.1. Callus Induction

**Figure 1:**
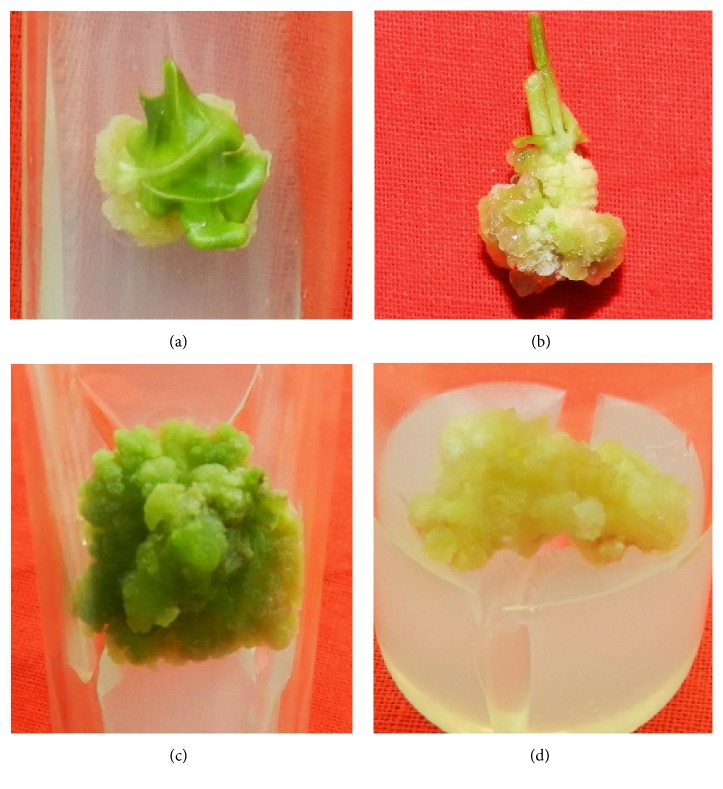
Friable callus induction from various explants of* R. tetraphylla*. (a) Friable white callus induced from leaf explants cultured on MS + NAA (2 mg/L). (b) Friable white callus induced from stem explants cultured on MS +Kn (1 mg/L). (c) Friable green callus induced from leaf explant cultured on MS+ Kn (3 mg/L). (d) Friable brown callus induced from root explant cultured on MS + 2,4-D (5 mg/L).

In this study leaf, stem, and root explants of* R. tetraphylla* cultured on MS basal medium did not produce any callus, but when cultured on MS medium supplemented with various concentrations of plant growth regulators (2,4-D, NAA, and Kn), copious amounts of friable callus were produced, after 4 weeks of culture (Figures 1(a)–1(d)). The produced callus is of different types, such as friable white, friable pale brown, and compact brown callus. At lower concentrations (1-2 mg/L) of 2,4-D, NAA, and Kn, all the explants have produced friable white callus (Figures 1(a) and 1(b)) whereas friable green callus (Figure 1(c)) to friable brown callus (Figure 1(d)) was produced from the optimum (3 mg/L) and higher concentrations (4-5 mg/L) of plant growth regulators (2,4-D, NAA, and Kn), respectively.

On 2,4-D supplemented MS media, maximum amount of mean fresh and dry weight (mg) of callus (378.7±0.67, 19.2±0.15), (241.5±0.34, 10.3±0.08), and (323.8±0.36, 15.5±0.12) was observed in leaf, stem, and root explants cultured on 5, 4, and 3 mg/L of concentrations, respectively, after 3 weeks of culture (Table 1). On NAA supplemented media, maximum amount of mean fresh and dry weight (mg) of callus (313.5±0.23, 19.5±0.27), (412.8±0.49, 25.1±0.15), and (277.4±0.14, 16.1±0.03) was observed in leaf, stem, and root explants cultured on 2, 6, and 2 mg/L concentrations, respectively, after 3 weeks of culture (Table 2). On Kn supplemented media, maximum amount of mean fresh and dry weight (mg) of callus (349.5±0.46, 22.8±0.19) and (343.2±0.64, 14.4±0.26) was observed in leaf and stem explants cultured on 5 mg/L concentration of Kn, after 3 weeks of culture (Table 3). Root explants have not responded on Kn supplemented media even after 4 weeks of culture.

Though the rate of callus growth was negligible in the first two weeks, the entire explant was covered with healthy callus after 3 weeks. Major portion of callus that developed from leaf, stem, and root was friable creamy white in color after 3 weeks of culture but became brown when cultured for more than six weeks on the same medium, due to the release of phenolic compounds, which is commonly observed in the callus cultures of shrub and tree species [30]. As the primarily induced callus of leaf and stem explants were friable and creamy white in color, in order to make them friable, green, and organogenic, the primary callus of leaf and stem explants of* R. tetraphylla* were subcultured for 3 passages of time (2 months) on the same combination of plant growth regulators.

Anitha and Kumari (2006a) [31] were first to report callus induction from leaf explants of* R. tetraphylla* on MS + 2,4-D (9 *μ*M/L) and the fresh and dry weights of callus were reported as 0.3354 and 0.0826 grams, respectively. Callus induction was also reported from petiole, cotyledonary leaf, and hypocotyl explants of* R. tetraphylla* cultured on MS medium supplemented with 4.52*μ*M of 2,4-D [32]. In our study, we have induced much higher value of mean fresh and dry weight (mg) of friable callus (378.7±0.67, 19.2±0.15) at 5 mg/L concentrations of 2,4-D from leaf explants. They have induced and used the callus cultures for reserpine estimation studies [31] (Anitha and Kumari 2006a) and studied the effect of NaCl amended media on callus growth [33].

From the above reports, it was clear that callus induced from various explants of* R. tetraphylla* was used for different studies by previous researchers but not for* in vitro* regeneration studies, whereas in our study we have utilized the induced callus for clonal multiplication of* R. tetraphylla* through* in vitro* regeneration studies.

### 3.2. Shoot Regeneration

**Figure 2:**
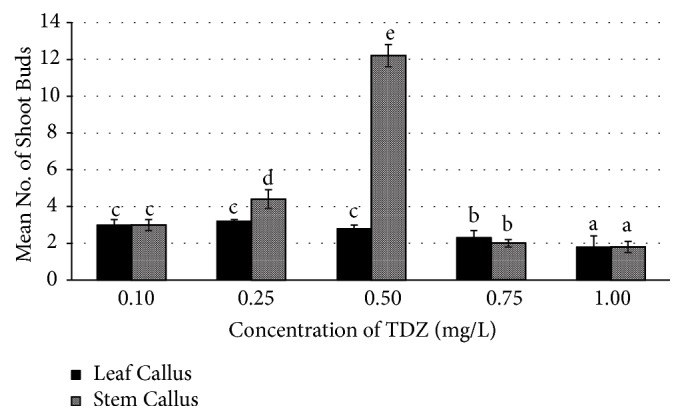
Effect of TDZ (Thidiazuron) on mean number of shoot buds induced in leaf and stem calluses of* R. tetraphylla*. Bars represent mean± SE and bars denoted by the same letter are not significantly different by Tukey's test used at* P *= 5% probability.

**Figure 3:**
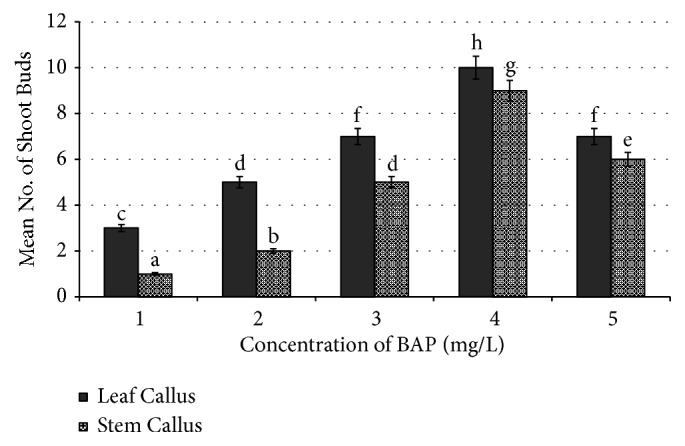
Effect of BAP (6-benzyl aminopurine) on number of shoot buds induced in leaf callus and stem callus of* R. tetraphylla.* Bars represent mean± SE and bars denoted by the same letter are not significantly different by Tukey's test used at* P *= 5% probability.

**Figure 4:**
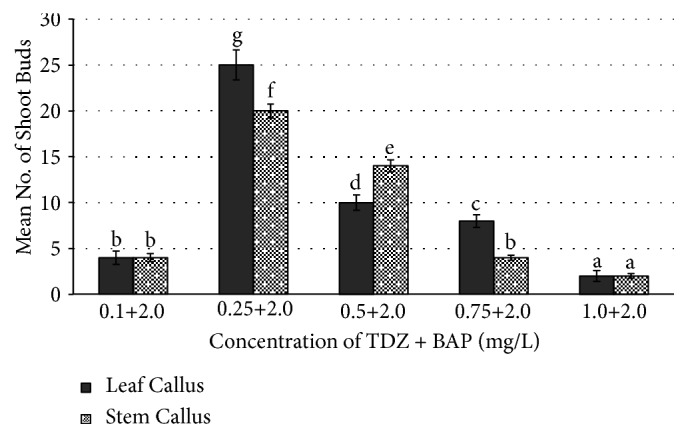
Effect of combination of TDZ and BAP on number of shoot buds induced from leaf callus and stem callus of* R. tetraphylla.* Bars represent mean± SE and bars denoted by the same letter are not significantly different by Tukey's test used at* P *= 5% probability.

In our earlier studies, we have used single cytokinin (TDZ) for regeneration of shoot buds from leaf and stem based callus and obtained maximum frequency of shoot regeneration (12.0±0.28) from stem based callus at 2.27 *μ*M/l concentration of TDZ [25]. Now through this research paper we are reporting a standardized protocol with much higher frequency of shoot regeneration from the friable callus of leaf and stem explants on MS media supplemented with individual (Figures 2 and 3) and combination of cytokinins (TDZ and BAP) (Figure 4).

**Figure 5:**
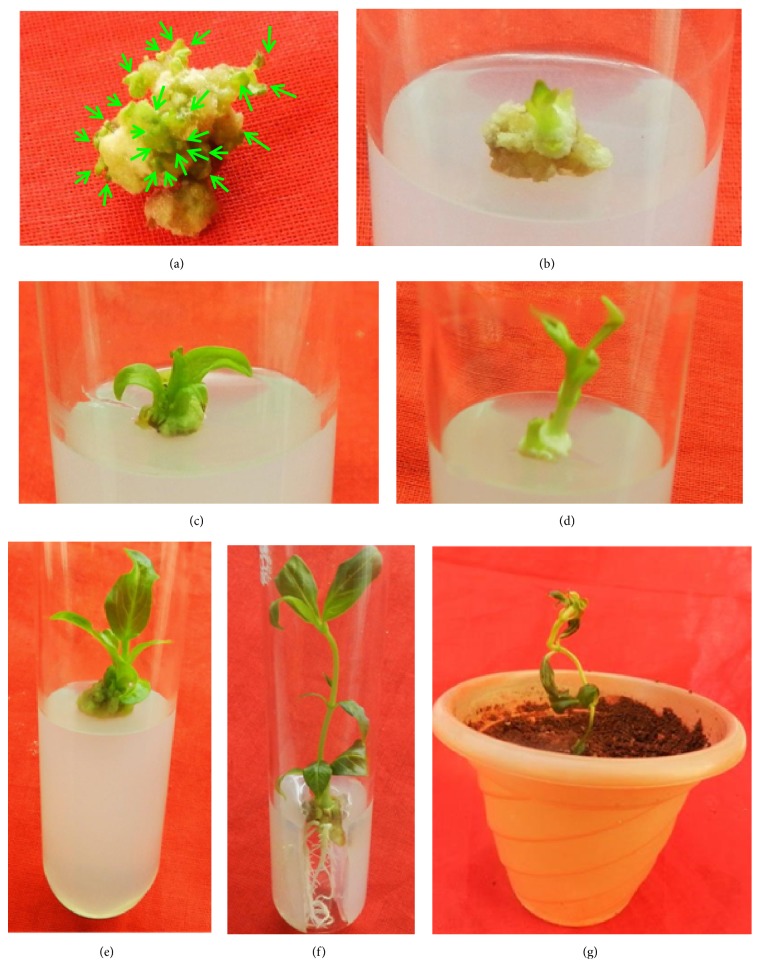
In vitro plant regeneration from leaf callus (3 times subcultured) in* R. tetraphylla*. (a) Shoot bud induction (represented with arrows) in leaf callus cultured on MS + TDZ (0.5 mg/L). (b) Transfer of individual micro shoots on MS basal media. (c) Single shoot elongation on MS basal medium. (d) Increase in shoot length up to 1 cm on MS basal medium after 1 week of culture. (e) Increase in shoot length up to 2 cm on MS basal medium after 2 weeks of culture. (f) Root induction in single shoot cultured on MS + IAA (1mg/L) + IBA (1 mg/L). (g) Complete plantlet transferred to plastic pot containing sterile soil for hardening.

**Figure 6:**
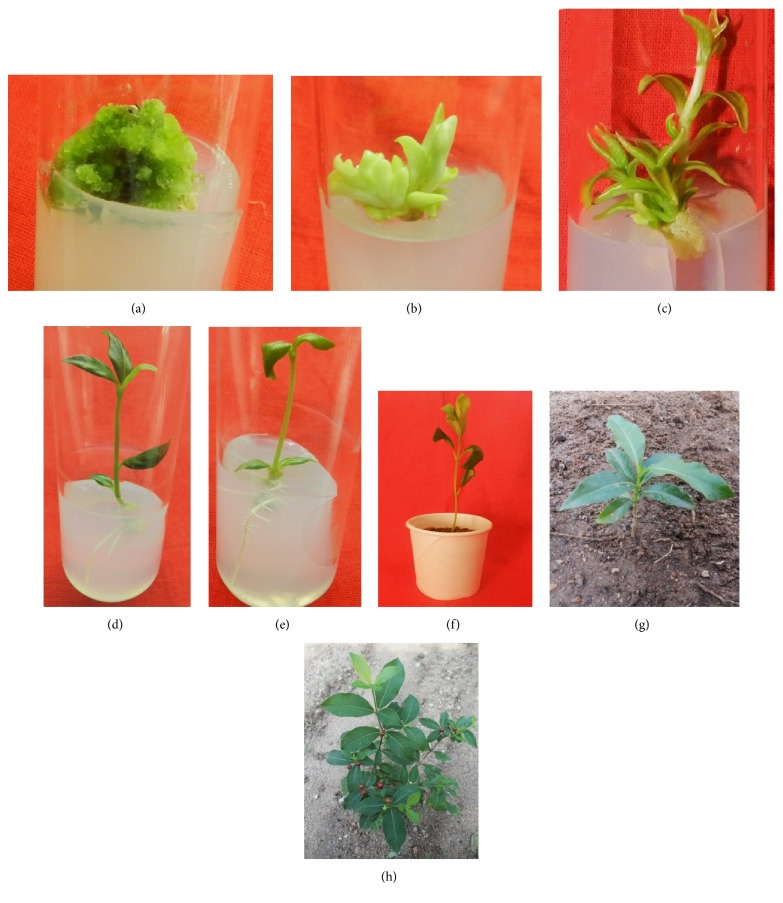
In vitro plant regeneration from stem callus (3 times sub cultured) in* R. tetraphylla.* (a) Shoot bud induction from stem callus cultured on MS + TDZ (0.25 mg/L) + BAP (2 mg/L). (b) Multiple shoot buds transferred onto MS basal media. (c) Elongation of multiple shoot buds into small shoot lets on MS basal media. (d) Root induction in single shoot cultured on MS + IAA (1mg/L) + IBA (1 mg/L). (e) Root induction in single shoot cultured on MS + IAA (1mg/L) + IBA (1 mg/L). (f) Complete plantlet transferred to plastic cup containing sterile soil for hardening. (g) Field transfer (2 months old). (h) Acclimatized plant (24 months old).

In this study, three times subcultured callus of leaf and stem explants of* R. tetraphylla* has shown good response of shoot regeneration with maximum number of shoot buds on combinational media (TDZ+BAP) rather than individually supplemented media (TDZ or BAP). On MS media amended with individual TDZ plant growth regulators, regeneration of shoot buds with a frequency of 3.2±0.1 and 12.2±0.28 was obtained at 0.25mg/l and 0.50 mg/l concentration from leaf and stem based callus, respectively (Figure 5(a)). On BAP amended media, regeneration of shoot buds with a frequency of 10.0±0.42 and 9.0±0.34 was obtained at 4.0 mg/l concentration from leaf and stem based callus, respectively. Overall, the maximum number of shoot buds with high frequency of regeneration (25±0.25 and 20±0.76) was obtained from leaf and stem calluses, respectively, when cultured on MS media supplemented with TDZ (0.25 mg/L) + BAP (2 mg/L), after 4 weeks of culture (Table 4 and Figure 6(a)).

Thidiazuron (TDZ) (N-phenyl-N′-1,2,3-thiadiazol-5-ylurea) is a thiadiazole-substituted phenylurea, which was reported to possess more cytokinin activity as compared to phenyl urea derivatives and other active adenine types of cytokinins [34]. Thidiazuron was also reported to possess actions of other plant growth regulators such as auxins, gibberellins, and ethylene [35]. Thidiazuron (TDZ) was used by several researchers in regeneration studies of medicinal plants with higher frequency of shoot let production from friable callus [36, 37]. In our earlier studies we have induced low frequency of multiple shoots from stem callus of* R. tetraphylla* on TDZ amended media [25].

It was reported that thidiazuron in combination with other plant growth regulators induced callus [38], somatic embryos [39], and shoot regeneration from friable callus [40]. Hence, in this study we have used TDZ in combination with BAP for the indirection regeneration of* R. tetraphylla*. In our study, combination of TDZ (0.25 mg/L) + BAP (2 mg/L) has resulted in inducing maximum number of shoot buds (25±0.25 and 20±0.76) from leaf and stem calluses, respectively, which is much higher compared to our earlier reports. TDZ+BAP combinations have been reported for successful multiple and rapid shoot regeneration of various species, e.g.,* Ancistrocladus heyneanus* [41],* Luffa cylindrical* L. [42],* Rauwolfia tetraphylla* L. [25],* Oryza sativa* [43],* Mirabilis jalapa *L. [44],* Achyranthes aspera* [40], and* Morus *Sps. cv. PPR-1 [26].

As the regenerated shoots are minute in the form of micro shoots, they were transferred onto MS basal media (Figures 5(b) and 6(b)) and cultured for 2 weeks for elongation of micro shoots into shoot lets (Figures 5(c), 5(d), 5(e), and 6(c)).

### 3.3. Rooting of In Vitro Regenerated Shoots

**Figure 7:**
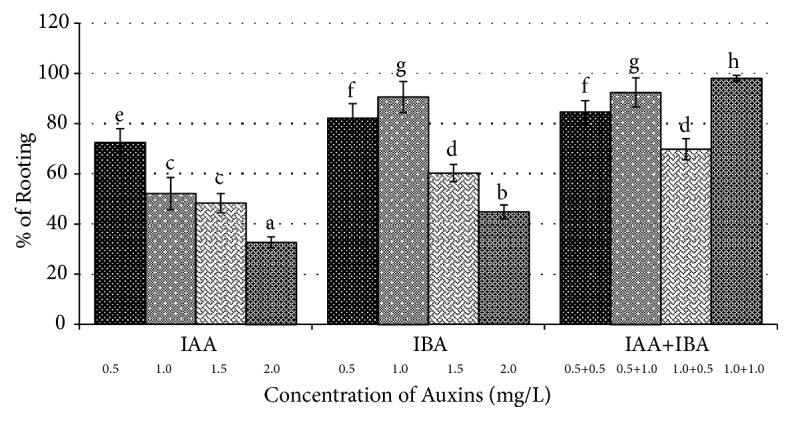
Effect of Auxins (IAA & IBA) on root induction from* in vitro* shoot lets of* Rauwolfia tetraphylla* L.

The regenerated shoots of* R. tetraphylla *L. were rooted successfully with maximum rooting frequency of 98.0±1.2 on combinational media of auxins with IAA (1.0 mg/L) and IBA (1.0 mg/L), after 2 weeks of culture, and well developed plantlet with extensive rooting was observed after 4 weeks of culture (Figures 5(f), 6(d), and 6(e)). On individual auxins supplemented media, rooting was induced with a frequency of 72.4±5.6 and 90.6±6.2 from IAA (0.5 mg/L) and IBA (1.0 mg/L), respectively (Table 5 and Figure 7). Rapid growth of plant lets was observed once the primary roots were initiated from the ends of shoot lets.

### 3.4. Plant Acclimatization to Ex Vitro Conditions

*In vitro *raised plantlets of* R. tetraphylla* were separated carefully from the media and hardened in a plastic pot (Figures 5(g) and 6(f)) using 2:1 ratio of sterile garden soil and sand. Initially the hardened plantlets were kept in hardened pots and cups for 4-6 weeks under controlled conditions by irrigating with broth solution of half strength MS salts and then transferred and acclimatized to field conditions (Figures 6(g) and 6(h)) with 86% survival. With garden soil and sand, similar types of results were obtained in acclimatizing the* in vitro* raised* Morus* Sps [45, 46].

### 3.5. Assessment of Genetic Stability Using SCoT, ISSR, and RAPD Markers

Genetic fidelity of micropropagated and acclimated plantlets of* R. tetraphylla* was assessed by using three generations of markers,* i.e.*, RAPD, ISSR, and SCoT. For better analysis of genetic homogeneity and for discarding the possibility of somaclonal variants, it was always recommended to use more than one marker while carrying out the genetic stability studies [47].

Analysis with SCoT primers (10 primers) has produced a total of 30 monomorphic bands and 3 as the average number of bands per primer with band size (in bp) ranging from 400 to 2000 (Table 6). Among the 10 SCoT primers, three primers, namely, S4, S5, and S6, have produced the highest number of bands (4 bands) and three primers, namely, S1, S2, and S9, have produced the least number of bands (2 bands) which were monomorphic to the mother plant. Amplification with 10 ISSR primers has yielded 45 bands in total with 4 as the average number of bands per primer and band size (in bp) ranging from 300 to 2500 (Table 7). ISSR primer, R10, has given good amplification and produced 6 DNA bands which are clear, distinct, and scorable, in the range (in bp) of 600-2500. Similarly, with 10 RAPD primers a total of 42 bands with 4 as the average number of bands per primer and band size (in bp) ranging from 300 to 2000 (Table 8) were obtained. RAPD primer, OPA3, has given good amplification and produced 6 DNA bands which are clear, distinct, and scorable, in the range (in bp) of 300-2200.

Even though we have carried amplification and produced monomorphic bands with RAPD primers, as we cannot ensure the reproducibility of results with RAPD primers, hence in our study along with RAPD primers we have also used other advanced primers,* i.e.*, ISSR and SCoT, which are reliable and reproducible type in assessing the genetic stability of regenerants compared to RAPD [48]. All amplification reactions with RAPD, ISSR, and SCoT primers were repeated three times to check the reproducibility and results were,* i.e.*, reproducible type with all the markers (R10-ISSR, S2-SCoT, and OPA3-RAPD).

Among the various markers, nowadays SCoT markers are gaining much attention due to their superiority and better resolvability over other markers [49, 50]. SCoT markers can be used effectively for both genetic diversity and similarity studies [51, 52]. SCoT markers were already reported for assessing the genetic stability of several plant species such as* Cleome gynandra* [53],* Pittosporum eriocarpum* [48]*, Albizia julibrissin *[54], and* Simmondsia chinensis *[55]. Similarly, ISSR primer based assessment of regenerans was reported in* Tylophora indica* [56],* Simmondsia chinensis *[57],* Rauwolfia tetraphylla* L. [58],* Spilanthes calva* [59],* Cucumis melo* L. [60],* Cornus alba* [61], and* Zea mays *[62].

**Figure 8:**
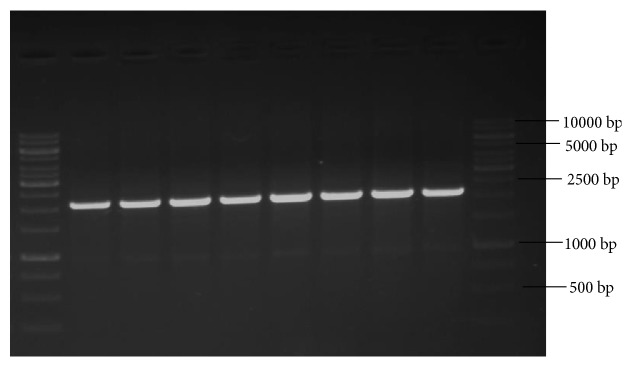
SCoT profiles of mother plant (Lane 2) and tissue culture raised plantlets (Lanes 3-9) of* R. tetraphylla* using S2-SCoT primer. Lanes 1 and 10: DNA ladder of 1Kbp size. Lane 2: DNA banding pattern of mother plant. Lanes 3-6: DNA banding pattern of acclimated plants which were raised from leaf explants. Lanes 7-9: DNA banding pattern of acclimated plants which were raised from stem explants.

**Figure 9:**
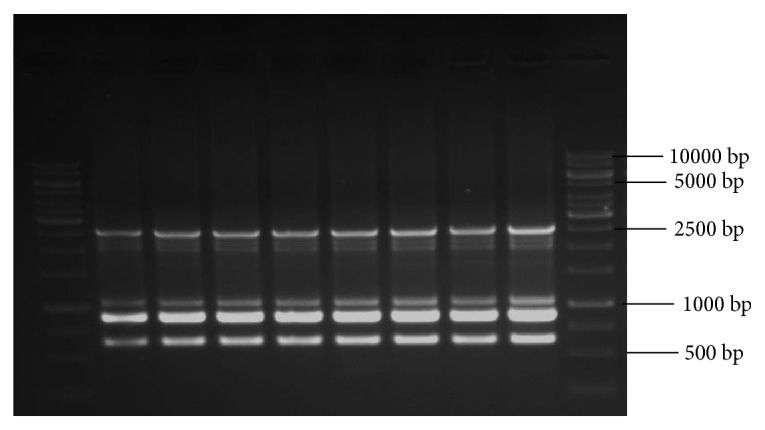
ISSR profiles of mother plant (Lane 2) and tissue culture raised plantlets (Lanes 3-9) of* R. tetraphylla* using R10-ISSR primer. Lanes 1 and 10: DNA ladder of 1Kbp size. Lane 2: DNA banding pattern of mother plant. Lanes 3-6: DNA banding pattern of acclimated plants which were raised from leaf explants. Lanes 7-9: DNA banding pattern of acclimated plants which were raised from stem explants.

**Figure 10:**
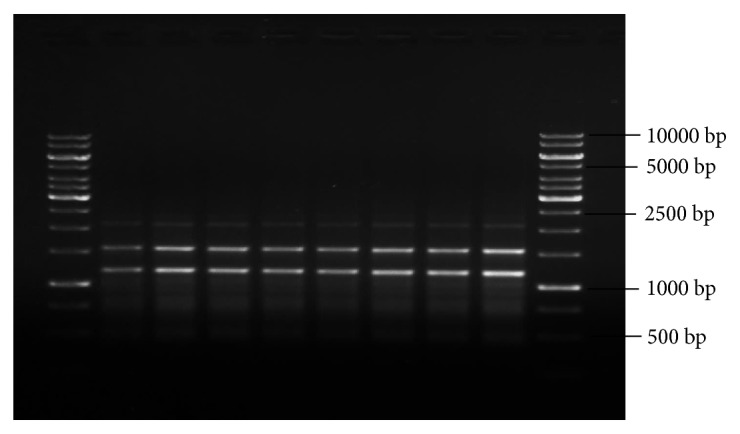
RAPD profiles of mother plant (Lane 2) and tissue culture raised plantlets (Lanes 3-9) of* R. tetraphylla* using OPA3-RAPD primer. Lanes 1 and 10: DNA ladder of 1Kbp size. Lane 2: DNA banding pattern of mother plant. Lanes 3-6: DNA banding pattern of acclimated plants which were raised from leaf explants. Lanes 7-9: DNA banding pattern of acclimated plants which were raised from stem explants.

In our study, DNA bands produced by amplification with RAPD, ISSR, and SCoT markers were monomorphic across the micro propagated plantlets and the mother plant (Figures 8, 9, and 10), which confirms the true-to-type and genetically stable nature of acclimated plantlets of* R. tetraphylla. *RAPD and ISSR primers were also used by earlier researchers for confirming the genetic homogeneity of regenerants in* R. tetraphylla* [25, 63, 64]. But, this is the first report of using SCoT primers for the assessment of genetic stability of regenerants in* R. tetraphylla*.

## 4. Conclusion

An efficient and repeatable protocol was developed for conservation of economically important plant species,* i.e.*,* R. tetraphylla *L., by high frequency of indirect shoot regeneration from leaf and stem based callus. Further, the genetic homogeneity of* in vitro *regenerated plantlets was confirmed by using SCoT, ISSR, and RAPD markers. This standardized protocol could be utilized for the mass propagation of true-to-type genotype of this endangered medicinal plant.

## Figures and Tables

**Table 1:** Effect of 2, 4-dichlorophenoxy acetic acid (2, 4-D), on callus induction from leaf, stem (internode), and root explants of *Rauwolfia tetraphylla* L.

Explant	MS + 2,4-D (mg/L)	Morphogenetic Response	Callus	
			Mean fresh weight (mg)	Mean dry weight (mg)
Leaf	1.0	Friable white callus	234.4 ± 0.12^i^	08.2 ± 0.44^k^
	2.0	Friable white callus	255.9 ± 0.23^g^	09.6 ± 0.24^j^
	3.0	Friable white callus	287.8 ± 0.91^f^	11.4 ± 0.49^h^
	4.0	Friable white callus	330.6 ± 0.76^b^	16.7 ± 0.68^b^
	5.0	Friable white callus	378.7 ± 0.67^a^	19.2 ± 0.15^a^

Stem (Inter node)	1.0	Friable white callus	92.6 ± 0.19^l^	1.03 ± 0.11^n^
	2.0	Friable white callus	123.5 ± 0.28^k^	2.30 ± 0.67^m^
	3.0	Friable white callus	185.6 ± 0.36^j^	06.6 ± 0.23^l^
	4.0	Friable white callus	241.5 ± 0.34^h^	12.3 ± 0.08^f^
	5.0	Compact brown callus	232.2 ± 0.24^i^	09.4 ± 0.24^j^

Root	1.0	Friable white callus	123.6 ± 0.34^k^	07.4 ± 0.55^k^
	2.0	Friable white callus	256.7 ± 0.12^i^	11.7 ± 0.23^g^
	3.0	Friable brown callus	323.8 ± 0.36^c^	15.5 ± 0.12^c^
	4.0	dark brown callus	294.3 ± 0.45^d^	14.6 ± 0.67^d^
	5.0	dark brown callus	288.3 ± 0.44^e^	13.8 ± 0.11^e^

Data represents average of three replicates. Each replicate consists of 25 cultures. Data scored at the end of 3 weeks of culture. Mean ± standard error. Mean followed by the same superscript in a column is not significantly different at *P*=0.05.

**Table 2:** Effect of naphthalene acetic acid (NAA), on callus induction from leaf, stem (internode), and root explants of *Rauwolfia tetraphylla* L.

Explant	MS + NAA (mg/L)	Morphogenetic Response	Callus	
			Mean fresh weight (mg)	Mean dry weight (mg)
Leaf	1.0	Friable white callus	311.7 ± 0.31^c^	19.1 ± 0.23^c^
	2.0	Friable white callus	313.5 ± 0.23^d^	19.5± 0.67^b^
	3.0	Friable pale brown callus with roots	303.6 ± 0.55^e^	19.0 ± 0.54^d^
	4.0	Friable pale brown callus with roots	291.5 ± 0.18^f^	17.2± 0.33^e^
	5.0	Friable pale brown callus with roots	242.4 ± 0.23^j^	15.6 ± 0.80^g^

Stem (Inter node)	1.0	Friable white callus	153.3 ± 0.14^l^	2.2± 0.43^m^
	2.0	Friable white callus	255.4 ± 0.40^i^	4.9 ± 0.45^l^
	3.0	Friable white callus	303.6 ± 0.47^e^	10.1 ± 0.13^h^
	4.0	Pale brown callus	343.1 ± 0.21^b^	15.8 ± 0.36^f^
	5.0	Pale brown callus	412.8 ± 0.49^a^	25.1 ± 0.15^a^

Root	1.0	White friable callus	256.5 ± 0.38^i^	9.5 ± 0.19^k^
	2.0	White friable callus	277.4 ± 0.14^g^	16.1 ± 0.03^f^
	3.0	Pale brown callus	262.1 ± 0.21^h^	14.6 ± 0.43^h^
	4.0	Brown callus	258.4 ± 0.74^i^	13.9 ± 0.67^i^
	5.0	Dark brown callus	243.8 ± 0.36^j^	13.2 ± 0.31^i^

Data represents average of three replicates. Each replicate consists of 25 cultures. Data scored at the end of 3 weeks of culture. Mean ± standard error. Mean followed by the same superscript in a column is not significantly different at *P*=0.05.

**Table 3:** Effect of Kinetin (Kn) on callus induction from leaf, stem (internode), and root explants of *Rauwolfia tetraphylla* L.

Explant	MS + Kn (mg/L)	Morphogenetic Response	Callus	
			Mean fresh weight (mg)	Mean dry weight (mg)
Leaf	1.0	No response	--	--
	2.0	Friable white callus	201.4 ± 0.11^h^	11.1 ± 0.14^f^
	3.0	Friable green callus	226.3 ± 0.41^f^	13.5 ± 0.22^d^
	4.0	Pale brown callus	334.6 ± 1.02^c^	18.5± 0.68^b^
	5.0	Pale brown callus	349.5 ± 0.46^a^	22.8 ± 0.19^a^

Stem (Inter node)	1.0	Friable white callus	218.6 ± 0.25^g^	3.6 ± 0.19^i^
	2.0	Friable White callus	233.3 ± 0.23^f^	4.2 ± 0.17^h^
	3.0	Friable White callus	264.9 ± 0.91^e^	7.8 ± 0.23^g^
	4.0	Pale brown callus	309.4 ± 0.87^d^	11.5 ± 0.61^e^
	5.0	Pale brown callus	343.2 ± 0.64^b^	14.4 ± 0.26^c^

Root	1.0	Single shoot bud	-	-
	2.0	Two shoot buds	-	-
	3.0	Multiple shoot buds	-	-
	4.0	Multiple shoot buds	-	-
	5.0	Multiple shoot buds	-	-

Data represents average of three replicates. Each replicate consists of 25 cultures. Data scored at the end of 3 weeks of culture. Mean ± standard error. Mean followed by the same superscript in a column is not significantly different at *P*=0.05.

**Table 4:** Effect of TDZ and BAP on shoot bud differentiation from 3 times subcultured leaf, stem, and root callusof *Rauwolfia tetraphylla* L.

Three times sub-cultured callus	TDZ (mg/L)	BAP (mg/L)	No. of shoot buds
Leaf	0.10	2.00	4 ± 0.12^f^
	0.25	2.00	25 ± 0.25^a^
	0.50	2.00	10 ± 0.17^d^
	0.75	2.00	8 ± 0.11^e^
	1.00	2.00	2 ± 0.15^g^

Stem	0.10	2.00	4 ± 0.43^f^
	0.25	2.00	20 ± 0.76^b^
	0.50	2.00	14 ± 0.13^c^
	0.75	2.00	4 ± 0.28^f^
	1.00	2.00	2 ± 0.26^g^

Data represents average of three replicates. Each replicate consists of 10 cultures. Data scored at the end of 2 weeks of culture. Mean ± standard error. Mean followed by the same superscript in a column is not significantly different at *P*=0.05.

**Table 5:** Effect of auxins (IAA and IBA) on root induction from *in vitro* shoot lets of *Rauwolfia tetraphylla* L.

IAA (mg/L)	IBA (mg/L)	Rooting (%)	No of roots/shoot
0.5	-	72.4 ± 5.6^e^	3.54 ± 0.62^c^
1.0	-	52.1 ± 6.4^c^	3.12 ± 0.24^c^
1.5	-	48.3 ± 3.8^b^	2.18 ± 0.28^b^
2.0	-	32.7 ± 2.2^a^	2.08 ± 0.13^b^

-	0.5	82.2 ± 5.7^f^	5.16 ± 0.44^e^
-	1.0	90.6 ± 6.2^g^	7.40 ± 0.72^g^
-	1.5	60.3 ± 3.4^d^	3.22 ± 0.84^c^
-	2.0	44.8 ± 2.8^b^	4.86 ± 0.32^d^

0.5	0.5	84.6 ± 4.6^f^	6.04 ± 0.54^f^
0.5	1.0	92.4 ± 5.8^g^	6.30 ± 0.28^f^
1.0	0.5	69.8 ± 4.2^d^	1.44 ± 0.12^a^
1.0	1.0	98.0 ± 1.2^h^	8.32 ± 0.84^h^

Data represents average of three replicates. Each replicate consists of 10 cultures. Data scored at the end of 2 weeks of culture. Mean ± standard error. Mean followed by the same superscript in a column is not significantly different at *P*=0.05.

**Table 6:** List of primers, their sequence, number and size of DNA bands generated by SCoT.

#	Primer code	Primer Sequence	Number of Bands	Band Length (in bp)
1	S1	CAACAATGGCTACCACCA	2	900-1200
2	S2	CAACAATGGCTACCACCC	2	1000-1500
3	S3	CAACAATGGCTACCACCG	3	1000-1400
4	S4	CAACAATGGCTACCACCT	4	500-2000
5	S5	CAACAATGGCTACCACGA	4	500-2000
6	S6	CAACAATGGCTACCACGC	4	500-2000
7	S7	CAACAATGGCTACCACGG	3	500-2000
8	S8	ACGACATGGCGACCAACG	3	400-2100
9	S9	ACCATGGCTACCACCGAC	2	500-1200
10	S10	CCATGGCTACCACCGCAG	3	400-1200

**Table 7:** List of primers, their sequence, number and size of DNA bands generated by ISSR.

#	Primer code	Primer Sequence	Number of Bands	Band Length (in bp)
1	R1	AGAGAGAGAGAGAGAGG	5	500-2000
2	R2	AGAGAGAGAGAGAGAGYT	5	750-2500
3	R3	AGAGAGAGAGAGAGAGTC	4	700-1700
4	R4	CTCTCTCTCTCTCTCTRA	4	500-2000
5	R5	GAGAGAGAGAGAGAGAT	6	400-2000
6	R6	GAGAGAGAGAGAGAGAC	4	500-200
7	R7	ACACACACACACACACT	3	750-1500
8	R8	CTCCTCCTCCTCCTCCTC	3	600-1100
9	R9	GAGAGAGAGAGAGAGAYT	6	300-2000
10	R10	GAGAGAGAGAGAGAGAYG	6	600-2500

**Table 8:** List of primers, their sequence, number and size of DNA bands generated by RAPD.

#	Primer code	Primer Sequence	Number of Bands	Band Length (in bp)
1	OPA1	CAGGCCCTTC	3	300-1500
2	OPA2	TGCCGAGCTG	5	300-1000
3	OPA3	AGTCAGCCAC	6	300-2200
4	OPA4	AATCGGGCTG	3	400-1200
5	OPA5	AGGGGTCTTG	4	500-1600
6	OPA6	TGGGCGTCAA	6	400-2000
7	OPA7	GGCATGACCT	5	300-1800
8	OPA8	TGGGCGTCAA	4	400-1900
9	OPA9	CCAGCAGCTT	3	600-1100
10	OPA10	GACTGCACAC	3	600-1200

## Data Availability

The data used to support the findings of this study are included within the article and can be freely available to authors with proper citation in their research work.

## References

[1] Dahanukar S., Thatte U. (2000). *Ayurveda Revisited, Popular Prakashan*.

[2] Chopra A., Doiphode V. V. (2002). Ayurvedic medicine: core concept, therapeutic principles, and current relevance. *Medical Clinics of North America*.

[3] Ahmedulla M., Nayar M. P. (1999). *Red data book of Indian Plants*.

[4] Sahu A. N., Hemalatha S., Sairam K. (2014). Phyto-Pharmacological review of Mesua ferrea Linn. *InternationalJournal of Phytology*.

[5] Lingaraj P. (2016). Medicinal Plants of India: With special reference to Odisha. *International Journal of Advanced Research In Ideas in Education*.

[6] Joshi K., Chavan P., Warude D., Patwardhan B. (2004). Molecular markers in herbal drug technology. *Current Science*.

[7] Singh K. M., Abhay K., Singh R. K. P., Ujjwal K. (2013). Medicinal and aromatic plants for enhancing farm income: the case of bihar. *Munich Personal Repec Archive*.

[8] Wiart C. (2006). *Medicinal Plants of Asia and the Pacific*.

[9] Rajkarnikar K. M., Sainju H. K., Bhatta B. D. In vitro culture of Rauwolfia serpentina L. benth. ex. Kurz.

[10] VAKIL R. J. (1949). A clinical trial of Rauwolfia serpentina in essential hypertension. *British Heart Journal*.

[11] Rohela G. K., Bylla P., Pendli S., Rajender K., Dharavath B., Thammidala C. R. (2018). ISSR marker based DNA profiling studies in Rauwolfia species. *Annals of Plant Sciences*.

[12] Chevalier A. (1996). *The Encyclopedia of Medicinal Plants*.

[13] Singh P., Singh A., Shukla A. K., Singh L., Pande V., Nailwal T. K. (2009). Somatic embryogenesis and in vitro regeneration of an endangered medicinal plant sarpgandha (rauvolfia serpentina l.). *Life Science Journal*.

[14] Faisal M., Ahmad N., Anis M. (2005). Shoot multiplication in Rauvolfia tetraphylla L. using thidiazuron. *Plant Cell, Tissue and Organ Culture*.

[15] Rohela G. K., Prasad B., Ravi C. H. (2015). In vitro clonal propagation of Rauwolfia tetraphylla, a relative of indian snake root plant. *Research Journal of Biotechnology*.

[16] Jyothi B., Penchala Pratap G., Sudharshan G. (2011). Ethnobotanical investigation of underground plant parts from Chittoor District, Andhra Pradesh, India. *Life Science Leaf Lets*.

[17] Panda S. K., Debajoyti D., Bichitra N. (2012). Phyto-pharmacognostical studies and quantitative determination of reserpine in different parts of Rauwolfia(Spp) of Eastern Odisha by UV Spectrocsopy Method. *Asian Journal of Plant Sciences and Research*.

[18] Bhattacharjee S. K. (1998). Handbook of Medicinal Plants, Pointer Publications. *India*.

[19] Brahmachari G., Mandal L. C., Gorai D., Mondal A., Sarkar S., Majhi S. (2011). A new labdane diterpene from Rauvolfia tetraphylla Linn. (Apocynaceae). *Journal of Chemical Research*.

[20] Ghosh K. C., Banerjee N. (2003). Influence of plant growth regulators on in vitro micropropagation of Rauwolfia tetraphylla L.. *Phytomorphology: An International Journal of Plant Morphology*.

[21] Faisal M., Anis M. (2005). Rapid in vitro propagation of Rauvolfia tetraphylla L. - An endangered medicinal plant. *Physiology and Molecular Biology of Plants*.

[22] Mitra G. C. (1976). Studies on the formation of viable and non-viable seeds in Rauwolfia serpentinabenth. *Indian Journal of Experimental Biology*.

[23] Harisharanraj R., Suresh K., Saravanababu S. (2009). Rapid Clonal Propagation Rauvolfia tetraphylla L. *Academic Journal of Plant Sciences*.

[24] Sarma D., Sarma S., Baruah A. (1999). Micropropagation and in vitro flowering of Rauvolfia tetraphylla; a potent source of anti-hypertension drugs. *Planta Medica*.

[25] Rohela G. K., Bylla P., Kota S., Abbagani S., Chithakari R., Reuben T. C. (2013). In vitro plantlet regeneration from leaf and stem calluses of Rauwolfia tetraphylla (R.canescens) and confirmation of genetic fidelity of plantlets using the ISSR PCR method. *Journal of Herbs, Spices & Medicinal Plants*.

[26] Rohela G. K., Jogam P., Shabnam A. A., Shukla P., Abbagani S., Ghosh M. K. (2018). In vitro regeneration and assessment of genetic fidelity of acclimated plantlets by using ISSR markers in PPR-1 (Morus sp.): An economically important plant. *Scientia Horticulturae*.

[27] Murashige T., Skoog F. (1962). A revised medium for rapid growth and bio assays with tobacco tissue cultures. *Physiologia Plantarum*.

[28] Doyle J. J., Doyle J. L. (1990). Isolation of plant DNA from fresh tissue. *Focus*.

[29] Bylla P., Gulab K. R., Radha T. (2013). DNA profiling of commercial chilli pepper (Capsicum annuum L.) varieties using random amplified polymorphic DNA (RAPD) markers. *African Journal of Biotechnology*.

[30] Rohela G. K., Bylla P., Korra R. (2016). Phytochemical screening and antimicrobial activity of leaf, stem, root and their callus extracts in Rauwolfia tetraphylla. *International Journal of Agriculture & Biology*.

[31] Anitha S., Ranjitha Kumari B. D. (2006). In vitro flowering in Rauvolfia tetraphylla L.. *Pakistan Journal of Biological Sciences*.

[32] Anitha S., Kumari D. R. (2012). In vitro callus culture in Rauvolfia tetraphylla L.: Indole alkaloid production. *Asian Journal of Plant Sciences*.

[33] Anitha S., Ranjitha Kumari B. D. (2006). Reserplne accumulation in NaCI treated calli of Rauvolfia tetraphylla L. *ScienceAsia*.

[34] Huetteman C. A., Preece J. E. (1993). Thidiazuron: a potent cytokinin for woody plant tissue culture. *Plant Cell, Tissue and Organ Culture*.

[35] Pai S. R., Desai N. S., Ahmad. N., Faisal M. (2018). Effect of TDZ on various plant cultures. *Thidiazuron: From urea derivative to plant growth regulator*.

[36] Ravi C. H., Avinash K. S., Gulab K. R. (2012). High frequency in vitro clonal propagation of Solanum surattense burm. F. *International Journal of Pharma and Biological Sciences*.

[37] Korra R., Bylla P., Rohela G. K. (2017). In vitro micro propagation and confirmation of genetic fidelity using RAPD marker in ethno medicinal plant Stachytarpheta jamaicensis L. Vahl. *International Journal of Advanced Research*.

[38] Azeez H., Ibrahim K., Pop R., Pamfil D., Hârţa M., Bobiș O. (2017). Changes induced by gamma ray irradiation on biomass production and secondary metabolites accumulation in Hypericum triquetrifolium Turra callus cultures. *Industrial Crops and Products*.

[39] Baskaran P., Van Staden J. (2017). Ultrastructure of somatic embryo development and plant propagation for Lachenalia montana. *South African Journal of Botany*.

[40] Pai S. R., Upadhya V., Hedge H. V., Joshi R. K., Kholkute S. D. (2018). In vitro rapid multiplication and determination of triterpenoids in callus cultures of Achyranthes aspera Linn. *Indian Journal of Biochemistry and Biophysics*.

[41] Pai S. R., Nimbalkar M. S., Pawar N. V., Kedage V. V., Dixit G. B. (2008). In vitro embryo culture and Ex situ regeneration studies in Ancistrocladus heyneanus Wall. Ex Grah. *Plant Cell Biotechnology and Molecular Biology*.

[42] Sujatha D., Ravi C. H., Raghuvardhanet L. (2013). In vitro plantlet regeneration and genetic transformation of sponge gourd (Luffa cylindrica L.). *African Journal of Plant Sciences*.

[43] Mohd Din A. R., Iliyas Ahmad F., Wagiran A., Abd Samad A., Rahmat Z., Sarmidi M. R. (2016). Improvement of efficient in vitro regeneration potential of mature callus induced from Malaysian upland rice seed (Oryza sativa cv. Panderas). *Saudi Journal of Biological Sciences*.

[44] Khan Rohela G., Damera S., Bylla P., Korra R., Pendli S., Thammidala C. (2016). Somatic embryogenesis and indirect regeneration in Mirabilis jalapa Linn.. *Materials Today: Proceedings*.

[45] Rohela G. K., Aftab A. S., Pawan S. (2018). In vitro clonal propagation of PPR-1, a superior temperate mulberry variety. *Indian Journal of Biotechnology*.

[46] Rohela G. K., Shabnam A. A., Shukla P., Kamili A. N., Ghosh M. K. (2018). Rapid one step protocol for the in vitro micro propagation of morus multicaulis var. goshoerami, an elite mulberry variety of temperate region. *Journal of Experimental Biology and Agricultural Sciences*.

[47] Lakshmanan V., Venkataramareddy S. R., Neelwarne B. (2007). Molecular analysis of genetic stability in long-term micropropagated shoots of banana using RAPD and ISSR markers. *Electronic Journal of Biotechnology*.

[48] Thakur J., Dwivedi M. D., Sourabh P., Uniyal P. L., Pandey A. K. (2016). Genetic homogeneity revealed using SCoT, ISSR and RAPD markers in micropropagated Pittosporum eriocarpum Royle- an endemic and endangered medicinal plant. *PLoS ONE*.

[49] Collard B. C. Y., Mackill D. J. (2009). Start Codon Targeted (SCoT) polymorphism: a simple, novel dna marker technique for generating gene-targeted markers in plants. *Plant Molecular Biology Reporter*.

[50] Gorji A. M., Poczai P., Polgar Z., Taller J. (2011). Efficiency of arbitrarily amplified dominant markers (SCOT, ISSR and RAPD) for diagnostic fingerprinting in tetraploid potato. *American Journal of Potato Research*.

[51] Cabo S., Ferreira L., Carvalho A., Martins-Lopes P., Martín A., Lima-Brito J. E. (2014). Potential of Start Codon Targeted (SCoT) markers for DNA fingerprinting of newly synthesized tritordeums and their respective parents. *Journal of Applied Genetics*.

[52] Fang-Yong C., Ji-Hong L. (2014). Germplasm genetic diversity of Myrica rubra in Zhejiang Province studied using inter-primer binding site and start codon-targeted polymorphism markers. *Scientia Horticulturae*.

[53] Rathore N. S., Rai M. K., Phulwaria M., Rathore N., Shekhawat N. S. (2014). Genetic stability in micropropagated Cleome gynandra revealed by SCoT analysis. *Acta Physiologiae Plantarum*.

[54] Rahmani M., Pijut P. M., Shabanian N., Nasri M. (2015). Genetic fidelity assessment of in vitro-regenerated plants of Albizia julibrissin using SCoT and IRAP fingerprinting. *In Vitro Cellular & Developmental Biology - Plant*.

[55] Bekheet S. A., Gabr A. M. M., Reda A. A., El-Bahr M. K. (2015). Micropropagation and assessment of genetic stability of In Vitro raised jojoba (Simmondsia chinensis Link.) plants using SCoT and ISSR markers. *Plant Tissue Culture and Biotechnology*.

[56] Sharma M. M., Verma R. N., Singh A., Batra A. (2014). Assessment of clonal fidelity of Tylophora indica (Burm. f.) Merrill “in vitro” plantlets by ISSR molecular markers. *Springer Plus*.

[57] Kumar S., Mangal M., Dhawan A. K., Singh N. (2011). Assessment of genetic fidelity of micropropagated plants of Simmondsia chinensis (Link) Schneider using RAPD and ISSR markers. *Acta Physiologiae Plantarum*.

[58] Faisal M., Alatar A. A., Ahmad N., Anis M., Hegazy A. K. (2012). An efficient and reproducible method for in vitro clonal multiplication of *Rauvolfia tetraphylla* L. and evaluation of genetic stability using DNA-based markers. *Applied Biochemistry and Biotechnology*.

[59] Razaq M., Heikrujam M., Chetri S. K., Agrawal V. (2013). In vitro clonal propagation and genetic fidelity of the regenerants of Spilanthes calva DC. using RAPD and ISSR marker. *Physiology and Molecular Biology of Plants*.

[60] Raji M. R., Lotfi M., Tohidfar M. (2018). Somatic embryogenesis of muskmelon (Cucumis melo L.) and genetic stability assessment of regenerants using flow cytometry and ISSR markers. *Protoplasma*.

[61] Ilczuk A., Jacygrad E. (2016). In vitro propagation and assessment of genetic stability of acclimated plantlets of Cornus alba L. using RAPD and ISSR markers. *In Vitro Cellular & Developmental Biology - Plant*.

[62] Ramakrishnan M., Ceasar S. A., Duraipandiyan V., Ignacimuthu S. (2014). Efficient plant regeneration from shoot apex explants of maize (Zea mays) and analysis of genetic fidelity of regenerated plants by ISSR markers. *Plant Cell, Tissue and Organ Culture*.

[63] Alatar A., Faisal M. (2012). Encapsulation of Rauvolfia tetraphylla microshoots as artificial seeds and evaluation of genetic fidelity using RAPD and ISSR markers. *Journal of Medicinal Plants Research*.

[64] Faisal M., Alatar A. A., Ahmad K. H. (2012). Molecular and biochemical characterization in rauvolfia tetraphylla plantlets grown from synthetic seeds following in vitro cold storage. *Applied Biochemistry and Biotechnology*.

